# Supplementary material for: Hair Dye and Relaxer Use among Cisgender Women in Embu and Nakuru Counties, Kenya: Associations with Perceived Risk of Breast Cancer and Other Health Effects
Source: Int J Environ Res Public Health. 2024 Jun 28;21(7):846. doi: 10.3390/ijerph21070846 (PMC11277196; doi:10.3390/ijerph21070846)

SUPPLEMENTARY FIGURE CAPTIONS

FIGURE S1. Adjusted odds ratios and 95% confidence intervals illustrating the association between perceptions about health effects and perceived breast cancer risk related to hair product use with current hair dye use among female study participants in Kenya, **overall and stratified by County.** Independent multivariable-adjusted logistic regression models were run with robust error variances to examine the association between perceptions about health effects and perceived breast cancer risk related to hair product use (strongly agree/strongly disagree to a perception vs. all other responses) with current hair dye use, overall and stratified by county (Embu vs. Nakuru). Current hair dye use is defined as the use of permanent and/or semi-permanent hair dye(s) in the past year. Each logistic regression model adjusted for relevant sociodemographic (age, education level, current occupation field, number of children under age 15 residing in household) and health history (age at menarche) characteristics. Associations were reported as adjusted odds ratios (ORs) and plotted as adjusted log odds ratios (log ORs), accompanied by their corresponding 95% confidence intervals (CIs). The Benjamini-Hochberg (B-H) approach was employed to correct for multiple comparisons, using a False Discovery Rate (FDR) of 0.05. Following B-H correction, associations with *P*-values ≤0.002 would be statistically significant, but none were.

FIGURE S2. Adjusted odds ratios and 95% confidence intervals illustrating the association between perceptions about health effects and perceived breast cancer risk related to hair product use with current relaxer use among female study participants in Kenya, **overall and stratified by County.** Independent multivariable-adjusted logistic regression models were run with robust error variances to examine the association between perceptions about health effects and perceived breast cancer risk related to hair product use (strongly agree/strongly disagree to a perception vs. all other responses) with current relaxer use, overall and stratified by county (Embu vs. Nakuru). Current relaxer use is defined as the use of chemical relaxer(s) or straightening product(s) in the past year. Each logistic regression model adjusted for relevant sociodemographic (age, education level, current occupation field, number of children under age 15 residing in household) and health history (age at menarche) characteristics. Associations were reported as adjusted odds ratios (ORs) and plotted as adjusted log odds ratios (log ORs), accompanied by their corresponding 95% confidence intervals (CIs). The Benjamini-Hochberg (B-H) approach was employed to correct for multiple comparisons, using a False Discovery Rate (FDR) of 0.05. Following B-H correction, associations with *P*-values ≤0.002 would be statistically significant, but none were.

TABLE S1. Perceptions about health effects and perceived breast cancer risk related to hair product use of female study participants in Kenya, overall and stratified by County

	OVERALL N=746					EMBU N=372					NAKURU N=374					EMBU VS. NAKURU	MISSING‡
N (%)	SD	D	N	A	SA	SD	D	N	A	SA	SD	D	N	A	SA	*P*†	N (%)
**PERCEPTIONS ABOUT HEALTH EFFECTS**																	
a. The personal hair care products I use affect my health.	156 (20.9)	317 (42.5)	82 (11.0)	144 (19.3)	41 (5.5)	80 (21.5)	135 (36.3)	38 (10.2)	85 (22.8)	30 (8.1)	76 (20.3)	182 (48.7)	44 (11.8)	59 (15.8)	11 (2.9)	<.001*	6 (0.8)
b. Organic, natural, non-toxic, or eco-friendly personal hair care products have fewer toxic chemicals than regular products.	30 (4.0)	87 (11.7)	91 (12.2)	359 (48.1)	174 (23.3)	19 (5.1)	45 (12.1)	47 (12.6)	178 (47.8)	81 (21.8)	11 (2.9)	42 (11.2)	44 (11.8)	181 (48.4)	93 (24.9)	.527	5 (0.7)
c. Consumers should be concerned about the health effects of personal hair care products.	15 (2.0)	32 (4.3)	17 (2.3)	358 (48.0)	313 (42.0)	7 (1.9)	17 (4.6)	7 (1.9)	178 (47.8)	162 (43.5)	8 (2.1)	15 (4.0)	10 (2.7)	180 (48.1)	151 (40.4)	.902	11 (1.5)
d. There is no reason to worry about the health effects from chemicals that might be in personal hair care products.	242 (32.4)	291 (39.0)	38 (5.1)	115 (15.4)	50 (6.7)	114 (30.6)	145 (39.0)	14 (3.8)	68 (18.3)	24 (6.5)	128 (34.2)	146 (39.0)	24 (6.4)	47 (12.6)	26 (7.0)	.120	10 (1.3)
e. Overall, the benefits of using personal hair care products outweigh any risks from exposure to toxic chemicals that might be in these products.	87 (11.7)	172 (23.1)	102 (13.7)	283 (37.9)	92 (12.3)	43 (11.6)	80 (21.5)	50 (13.4)	138 (37.1)	55 (14.8)	44 (11.8)	92 (24.6)	52 (13.9)	145 (38.8)	37 (9.9)	.335	10 (1.3)
f. Government agencies in Kenya do a good job of regulating personal care products to ensure they are safe for consumers.	73 (9.8)	92 (12.3)	63 (8.4)	299 (40.1)	209 (28.0)	34 (9.1)	34 (9.1)	26 (7.0)	139 (37.4)	131 (35.2)	39 (10.4)	58 (15.5)	37 (9.9)	160 (42.8)	78 (20.9)	<.001*	10 (1.3)
**PERCEIVED BREAST CANCER RISK[a]**																	
a. I am concerned that the labels of hair care products do NOT list all the ingredients.	65 (8.7)	125 (16.8)	85 (11.4)	313 (42.0)	153 (20.5)	46 (12.4)	55 (14.8)	39 (10.5)	156 (41.9)	73 (19.6)	19 (5.1)	70 (18.7)	46 (12.3)	157 (42.0)	80 (21.4)	.008*	5 (0.7)
b. Because I am concerned about breast cancer, I plan to go natural (style my hair without chemicals).	40 (5.4)	127 (17.0)	92 (12.3)	272 (36.5)	210 (28.2)	24 (6.5)	60 (16.1)	37 (9.9)	139 (37.4)	107 (28.8)	16 (4.3)	67 (17.9)	55 (14.7)	133 (35.6)	103 (27.5)	.227	5 (0.7)
c. Because I am concerned about breast cancer, I intend to watch the ingredients of the hair care products I use.	12 (1.6)	25 (3.4)	41 (5.5)	339 (45.4)	324 (43.4)	6 (1.6)	10 (2.7)	13 (3.5)	179 (48.1)	161 (43.3)	6 (1.6)	15 (4.0)	28 (7.5)	160 (42.8)	163 (43.6)	.109	5 (0.7)
d. All women should worry about the ingredients in hair products.	14 (1.9)	31 (4.2)	28 (3.8)	327 (43.8)	338 (45.3)	5 (1.3)	13 (3.5)	12 (3.2)	157 (42.2)	180 (48.4)	9 (2.4)	18 (4.8)	16 (4.3)	170 (45.5)	158 (42.2)	.349	8 (1.1)
e. Because I am concerned about breast cancer, I plan to adjust how I use hair care products.	11 (1.5)	28 (3.8)	49 (6.6)	367 (49.2)	283 (37.9)	6 (1.6)	17 (4.6)	20 (5.4)	176 (47.3)	149 (40.1)	5 (1.3)	11 (2.9)	29 (7.8)	191 (51.1)	134 (35.8)	.351	8 (1.1)
f. I want to learn more about the risk hair products can cause to my health.	8 (1.1)	4 (0.5)	9 (1.2)	268 (35.9)	449 (60.2)	6 (1.6)	3 (0.8)	2 (0.5)	123 (33.1)	234 (62.9)	2 (0.5)	1 (0.3)	7 (1.9)	145 (38.8)	215 (57.5)	.079	8 (1.1)

Abbreviations: SD, Strongly Disagree; D, Disagree; N, Neither Agree nor Disagree; A, Agree; SA, Strongly Agree.

† For perceptions about health effects and perceived breast cancer risk related to hair product use, comparisons of proportions of Likert scale responses across counties were assessed using the two-sided Chi-Squared test. The Benjamini-Hochberg (B-H) approach was employed to correct for multiple comparisons, using a False Discovery Rate (FDR) of 0.05. Following B-H correction, comparisons with *P*-values ≤0.008 (marked with an asterisk) remained statistically significant.

‡ The count and proportion missing pertain to the overall sample.

[a] 6-item subscale from the Black Identity, Hair Product Use, and Breast Cancer Scale (BHBS) on perceived breast cancer risk related to hair product use.

TABLE S2. Characteristics of female study participants in the overall sample who perceived health effects and breast cancer risk to be related to hair product use

CHARACTERISTICS	TOTAL N (%)	PERCEPTIONS ABOUT HEALTH EFFECTS related to hair product use N (%)						PERCEIVED BREAST CANCER RISK related to hair product use[a] N (%)					
		a. STRONGLY AGREE that the personal hair care products I use affect my health.	b. STRONGLY AGREE that organic, natural, non-toxic, or eco-friendly personal hair care products have fewer toxic chemicals than regular products.	c. STRONGLY AGREE that consumers should be concerned about the health effects of personal hair care products.	d. STRONGLY DISAGREE that there is no reason to worry about the health effects from chemicals that might be in personal hair care products.	e. STRONGLY DISAGREE that, overall, the benefits of using personal hair care products outweigh any risks from exposure to toxic chemicals that might be in these products.	f. STRONGLY DISAGREE that government agencies in Kenya do a good job of regulating personal care products to ensure they are safe for consumers.	a. STRONGLY AGREE that I am concerned that the labels of hair care products do NOT list all the ingredients.	b. STRONGLY AGREE that because I am concerned about breast cancer, I plan to go natural (style my hair without chemicals).	c. STRONGLY AGREE that because I am concerned about breast cancer, I intend to watch the ingredients of the hair care products I use.	d. STRONGLY AGREE that all women should worry about the ingredients in hair products.	e. STRONGLY AGREE that because I am concerned about breast cancer, I plan to adjust how I use hair care products.	f. STRONGLY AGREE that I want to learn more about the risk hair products can cause to my health.
**OVERALL**	**746 (100.0)**	**41 (5.5)**	**174 (23.3)**	**313 (42.0)**	**242 (32.4)**	**87 (11.7)**	**73 (9.8)**	**153 (20.5)**	**210 (28.2)**	**324 (43.4)**	**338 (45.3)**	**283 (37.9)**	**449 (60.2)**
**Age (years), *mean±SD***	30.4±8.1	32.1±8.4	30.9±8.1	30.2±8.2	30.6±8.2	31.1±8.5	31.5±8.9	30.2±8.5	31.2±8.7	29.9±8.0	30.4±8.3	30.2±8.4	30.5±8.2
**Education level**													
Less than high school certificate	142 (19.0)	11 (26.8)	38 (21.8)	45 (14.4)	40 (16.5)	23 (26.4)	18 (24.7)	28 (18.3)	52 (24.8)	67 (20.7)	64 (18.9)	49 (17.3)	86 (19.2)
High school certificate	283 (37.9)	17 (41.5)	72 (41.4)	120 (38.3)	91 (37.6)	32 (36.8)	22 (30.1)	60 (39.2)	83 (39.5)	114 (35.2)	119 (35.2)	108 (38.2)	169 (37.6)
Some college but no degree	253 (33.9)	11 (26.8)	49 (28.2)	114 (36.4)	92 (38.0)	25 (28.7)	28 (38.4)	49 (32.0)	61 (29.0)	111 (34.3)	119 (35.2)	96 (33.9)	150 (33.4)
Bachelor's degree and above[b]	67 (9.0)	2 (4.9)	15 (8.6)	34 (10.9)	19 (7.9)	7 (8.0)	5 (6.8)	16 (10.5)	14 (6.7)	32 (9.9)	36 (10.7)	30 (10.6)	44 (9.8)
**Current occupation field**													
Business and Administration	155 (20.8)	11 (26.8)	41 (23.6)	72 (23.0)	50 (20.7)	15 (17.2)	10 (13.7)	34 (22.2)	57 (27.1)	70 (21.6)	75 (22.2)	68 (24.0)	95 (21.2)
Farming and Agriculture	60 (8.0)	5 (12.2)	11 (6.3)	20 (6.4)	13 (5.4)	10 (11.5)	10 (13.7)	12 (7.8)	22 (10.5)	26 (8.0)	32 (9.5)	28 (9.9)	35 (7.8)
Sales and Service[c]	364 (48.8)	15 (36.6)	81 (46.6)	143 (45.7)	118 (48.8)	37 (42.5)	37 (50.7)	74 (48.4)	82 (39.0)	150 (46.3)	149 (44.1)	120 (42.4)	209 (46.5)
Student, Unemployed, or Casual Work	110 (14.7)	6 (14.6)	22 (12.6)	53 (16.9)	34 (14.0)	17 (19.5)	11 (15.1)	25 (16.3)	33 (15.7)	53 (16.4)	51 (15.1)	43 (15.2)	73 (16.3)
Other[d]	51 (6.8)	4 (9.8)	19 (10.9)	24 (7.7)	25 (10.3)	8 (9.2)	5 (6.8)	8 (5.2)	16 (7.6)	25 (7.7)	30 (8.9)	23 (8.1)	35 (7.8)
**Number of children under age 15 residing in household**													
0	186 (24.9)	10 (24.4)	44 (25.3)	89 (28.4)	61 (25.2)	21 (24.1)	23 (31.5)	42 (27.5)	50 (23.8)	89 (27.5)	85 (25.1)	71 (25.1)	106 (23.6)
1	242 (32.4)	17 (41.5)	50 (28.7)	104 (33.2)	76 (31.4)	25 (28.7)	24 (32.9)	54 (35.3)	68 (32.4)	101 (31.2)	111 (32.8)	98 (34.6)	153 (34.1)
2	210 (28.2)	7 (17.1)	54 (31.0)	83 (26.5)	74 (30.6)	25 (28.7)	15 (20.5)	38 (24.8)	55 (26.2)	89 (27.5)	91 (26.9)	71 (25.1)	125 (27.8)
≥3	99 (13.3)	7 (17.1)	24 (13.8)	33 (10.5)	27 (11.2)	15 (17.2)	11 (15.1)	17 (11.1)	33 (15.7)	41 (12.7)	46 (13.6)	40 (14.1)	60 (13.4)
**Age at menarche (years), *mean±SD***	14.3±1.7	14.2±2.0	14.1±1.6	14.2±1.6	14.3±1.7	14.5±1.7	14.5±1.6	14.4±1.9	14.3±1.9	14.2±1.7	14.1±1.7	14.2±1.7	14.2±1.7

[a] 6-item subscale from the Black Identity, Hair Product Use, and Breast Cancer Scale (BHBS).
[b] Individuals who completed a bachelor's degree and above (N=67, 9.0%) primarily comprised of those who completed a bachelor's degree (N=65, 8.7%), with the exception of two (0.3%) individuals who completed a master's degree in Embu County.
[c] Individuals in sales and service (N=364, 48.8%) largely comprised of cosmetologists (N=302, 40.5%).
[d] The other category (N=51, 6.8%) combines individuals who belong to the following occupation fields: education, government, law enforcement, science, technology, and engineering.

TABLE S3. Association between sociodemographic and health history characteristics with current hair dye (permanent and/or semi-permanent) and current relaxer use, overall and stratified by County

CURRENT USE OF HAIR PRODUCT	OVERALL				EMBU				NAKURU			
	Hair Dye		Relaxer		Hair Dye		Relaxer		Hair Dye		Relaxer	
n/N (%)	232/746 (31.1%)		266/746 (35.7%)		102/372 (27.4%)		141/372 (37.9%)		130/374 (34.8%)		125/374 (33.4%)	
	OR (95% CI)	*P* †	OR (95% CI)	*P* †	OR (95% CI)	*P* †	OR (95% CI)	*P* †	OR (95% CI)	*P* †	OR (95% CI)	*P* †
**Age (years)**	1.05 (1.02, 1.07)	<.001*	1.02 (1.00, 1.05)	.024	1.04 (1.00, 1.07)	.033	1.01 (0.98, 1.04)	.649	1.06 (1.02, 1.09)	.001*	1.05 (1.01, 1.08)	.004
**Education level**												
Less than high school certificate	ref.		ref.		ref.		ref.		ref.		ref.	
High school certificate	1.53 (0.95, 2.45)	.081	1.36 (0.85, 2.16)	.197	1.60 (0.82, 3.12)	.167	1.28 (0.68, 2.42)	.441	1.43 (0.70, 2.92)	.321	1.50 (0.74, 3.05)	.259
Some college but no degree	1.79 (1.08, 2.97)	.023	1.71 (1.03, 2.82)	.038	1.31 (0.63, 2.70)	.473	1.47 (0.72, 2.98)	.287	2.03 (0.96, 4.33)	.066	2.22 (1.03, 4.75)	.041
Bachelor's degree and above[a]	1.19 (0.57, 2.48)	.647	1.42 (0.70, 2.86)	.330	1.75 (0.58, 5.33)	.322	0.95 (0.34, 2.63)	.921	0.76 (0.26, 2.28)	.628	2.47 (0.88, 6.92)	.085
**Current occupation field**												
Business and Administration	ref.		ref.		ref.		ref.		ref.		ref.	
Farming and Agriculture	0.55 (0.24, 1.23)	.146	0.74 (0.35, 1.55)	.421	0.63 (0.21, 1.87)	.401	0.62 (0.24, 1.61)	.322	0.41 (0.11, 1.52)	.181	1.05 (0.32, 3.48)	.940
Sales and Service[b]	2.00 (1.30, 3.08)	.002*	2.07 (1.35, 3.16)	.001*	2.47 (1.34, 4.55)	.004	2.18 (1.25, 3.80)	.006	1.64 (0.86, 3.15)	.135	2.48 (1.19, 5.15)	.015
Student, Unemployed or Casual Work	1.50 (0.82, 2.76)	.190	1.62 (0.90, 2.90)	.106	1.11 (0.47, 2.62)	.815	1.14 (0.53, 2.43)	.741	2.03 (0.81, 5.07)	.132	2.92 (1.12, 7.56)	.028
Other[c]	1.21 (0.56, 2.62)	.625	1.14 (0.54, 2.40)	.722	1.38 (0.39, 4.82)	.618	1.60 (0.55, 4.68)	.389	0.88 (0.31, 2.50)	.816	1.01 (0.32, 3.21)	.982
**Number of children under age 15 residing in household**												
0	ref.		ref.		ref.		ref.		ref.		ref.	
1	1.03 (0.67, 1.59)	.891	1.24 (0.81, 1.90)	.318	0.92 (0.49, 1.74)	.806	1.00 (0.57, 1.78)	.994	1.09 (0.58, 2.06)	.781	1.69 (0.86, 3.33)	.130
2	0.86 (0.54, 1.36)	.514	1.13 (0.73, 1.76)	.574	0.92 (0.46, 1.82)	.807	0.93 (0.51, 1.72)	.828	0.71 (0.37, 1.35)	.296	1.53 (0.77, 3.04)	.220
≥3	1.01 (0.58, 1.77)	.962	1.12 (0.65, 1.93)	.693	2.43 (0.96, 6.14)	.061	1.40 (0.59, 3.30)	.442	0.54 (0.25, 1.17)	.119	1.26 (0.56, 2.82)	.573
**Age at menarche (years)**	1.04 (0.95, 1.14)	.429	1.02 (0.93, 1.12)	.649	1.15 (1.01, 1.30)	.035	1.07 (0.94, 1.21)	.296	0.91 (0.79, 1.06)	.234	0.96 (0.84, 1.10)	.562

† Independent logistic regression models were run with robust error variances to obtain odds ratios (ORs) with corresponding 95% confidence intervals (CIs) that examined the association between sociodemographic and health history characteristics of study participants with current hair dye use and current relaxer use, separately, in the overall sample and stratified by county (Embu vs. Nakuru). Current hair dye use is defined as the use of permanent and/or semi-permanent hair dye(s) in the past year. Current relaxer use is defined as the use of chemical relaxer(s) or straightening product(s) in the past year. The Benjamini-Hochberg (B-H) approach was employed to correct for multiple comparisons, using a False Discovery Rate (FDR) of 0.05. Following B-H correction, associations with *P*-values ≤0.002 (marked with an asterisk) remained statistically significant.

[a] Individuals who completed a bachelor's degree and above (overall: N=67, 9.0%) primarily comprised of those who completed a bachelor's degree (overall: N=65, 8.7%), with the exception of two (0.3%) individuals who completed a master's degree in Embu county.

[b] Individuals in sales and service (N) largely comprised of cosmetologists (n) – overall n/N: 302/364 (83.0%); Embu n/N: 112/155 (72.3%); Nakuru n/N: 190/209 (90.9%).

[c] The other category combines individuals who belong to the following occupation fields: education, government, law enforcement, science, technology, and engineering.

FIGURE S1.

CURRENT HAIR DYE USE

n/N (%)

OVERALL 232/746 (31.1%)

PERCEPTIONS ABOUT HEALTH EFFECTS	OR (95% CI)	Log OR (95% CI)	*P*
a. Strongly agree that the personal hair care products I use affect my health.	0.95 (0.48, 1.88)	-0.06 (-0.74, 0.63)	.874
b. Strongly agree that organic, natural, non-toxic, or eco-friendly personal hair care products have fewer toxic chemicals than regular products.	1.21 (0.77, 1.91)	0.19 (-0.27, 0.64)	.416
c. Strongly agree that consumers should be concerned about the health effects of personal hair care products.	1.20 (0.82, 1.77)	0.18 (-0.20, 0.57)	.351
d. Strongly disagree that there is no reason to worry about the health effects from chemicals that might be in personal hair care products.	1.20 (0.82, 1.75)	0.18 (-0.20, 0.56)	.354
e. Strongly disagree that, overall, the benefits of using personal hair care products outweigh any risks from exposure to toxic chemicals that might be in these products.	1.00 (0.58, 1.72)	0.00 (-0.54, 0.54)	.998
f. Strongly disagree that government agencies in Kenya do a good job of regulating personal care products to ensure they are safe for consumers.	1.03 (0.59, 1.77)	0.03 (-0.52, 0.57)	.927
**PERCEIVED BREAST CANCER RISK**			
a. Strongly agree that I am concerned that the labels of hair care products do NOT list all the ingredients.	1.00 (0.65, 1.53)	0.00 (-0.42, 0.43)	.998
b. Strongly agree that because I am concerned about breast cancer, I plan to go natural (style my hair without chemicals).	0.84 (0.55, 1.29)	-0.17 (-0.60, 0.26)	.437
c. Strongly agree that because I am concerned about breast cancer, I intend to watch the ingredients of the hair care products I use.	0.77 (0.48, 1.25)	-0.26 (-0.73, 0.22)	.293
d. Strongly agree that all women should worry about the ingredients in hair products.	1.07 (0.68, 1.68)	0.07 (-0.38, 0.52)	.768
e. Strongly agree that because I am concerned about breast cancer, I plan to adjust how I use hair care products.	1.07 (0.69, 1.66)	0.07 (-0.37, 0.51)	.762
f. Strongly agree that I want to learn more about the risk hair products can cause to my health.	1.37 (0.88, 2.13)	0.31 (-0.13, 0.76)	.164

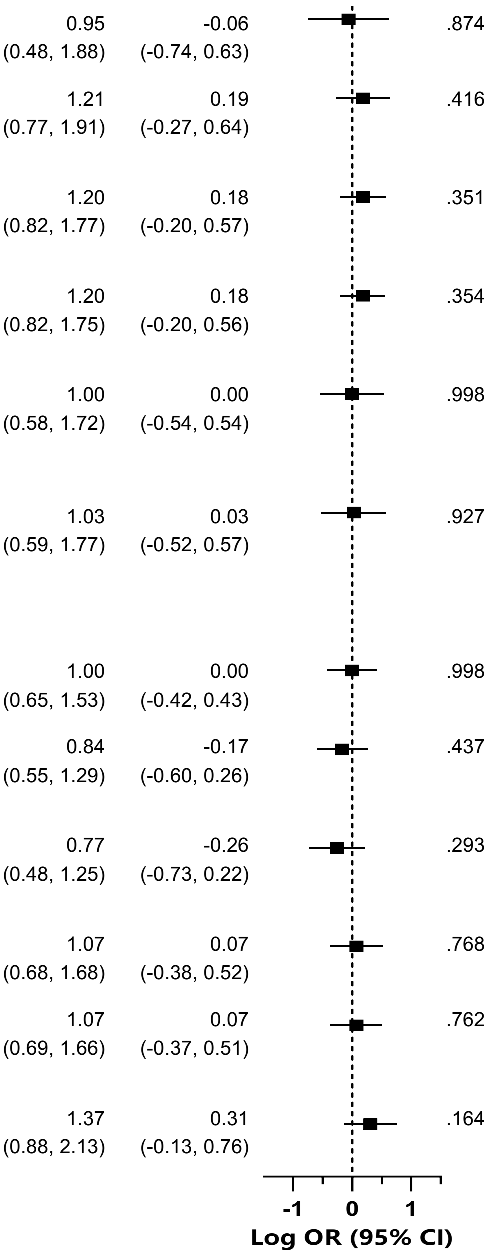

EMBU 102/372 (27.4%)

Item	OR (95% CI)	Log OR (95% CI)	*P*
Health effects a.	0.93 (0.38, 2.26)	-0.08 (-0.97, 0.82)	.866
Health effects b.	1.12 (0.54, 2.33)	0.11 (-0.62, 0.85)	.766
Health effects c.	1.31 (0.74, 2.33)	0.27 (-0.30, 0.85)	.349
Health effects d.	1.48 (0.84, 2.60)	0.39 (-0.17, 0.95)	.173
Health effects e.	0.86 (0.38, 1.96)	-0.15 (-0.98, 0.67)	.720
Health effects f.	0.97 (0.39, 2.42)	-0.03 (-0.94, 0.88)	.956
Breast cancer risk a.	1.36 (0.71, 2.61)	0.31 (-0.34, 0.96)	.349
Breast cancer risk b.	0.70 (0.37, 1.31)	-0.36 (-0.99, 0.27)	.260
Breast cancer risk c.	0.48 (0.23, 1.03)	-0.72 (-1.48, 0.03)	.061
Breast cancer risk d.	1.44 (0.65, 3.19)	0.37 (-0.43, 1.16)	.365
Breast cancer risk e.	0.83 (0.41, 1.69)	-0.18 (-0.89, 0.52)	.612
Breast cancer risk f.	1.84 (0.89, 3.78)	0.61 (-0.11, 1.33)	.098

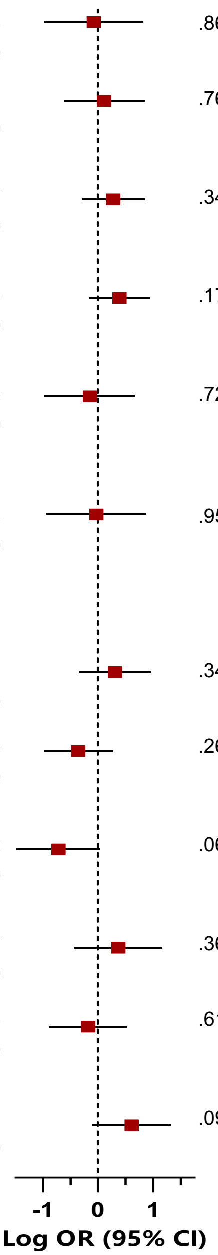

NAKURU 130/374 (34.8%)

Item	OR (95% CI)	Log OR (95% CI)	*P*
Health effects a.	1.45 (0.42, 4.98)	0.37 (-0.86, 1.61)	.551
Health effects b.	1.35 (0.73, 2.48)	0.30 (-0.31, 0.91)	.341
Health effects c.	1.08 (0.63, 1.85)	0.08 (-0.47, 0.62)	.785
Health effects d.	0.93 (0.55, 1.57)	-0.08 (-0.60, 0.45)	.777
Health effects e.	0.98 (0.47, 2.05)	-0.02 (-0.76, 0.72)	.959
Health effects f.	0.98 (0.48, 2.01)	-0.02 (-0.73, 0.70)	.964
Breast cancer risk a.	0.84 (0.47, 1.53)	-0.17 (-0.76, 0.42)	.576
Breast cancer risk b.	0.84 (0.44, 1.60)	-0.18 (-0.82, 0.47)	.593
Breast cancer risk c.	0.93 (0.49, 1.75)	-0.07 (-0.70, 0.56)	.822
Breast cancer risk d.	1.07 (0.59, 1.94)	0.06 (-0.53, 0.66)	.834
Breast cancer risk e.	1.41 (0.77, 2.59)	0.35 (-0.26, 0.95)	.265
Breast cancer risk f.	1.05 (0.58, 1.92)	0.05 (-0.55, 0.65)	.872

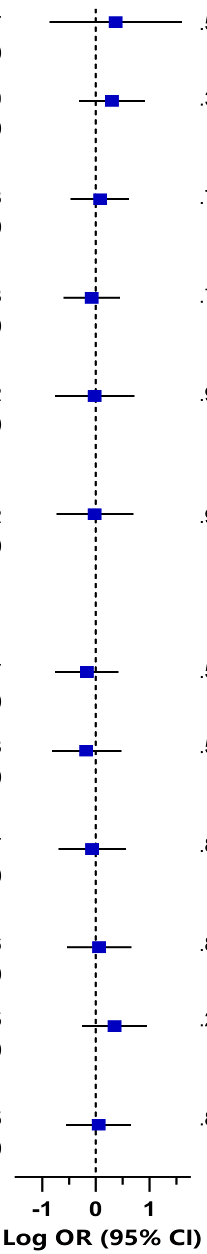

FIGURE S2.

CURRENT RELAXER USE	OVERALL			EMBU			NAKURU		
n/N (%)	266/746 (35.7%)			141/372 (37.9%)			125/374 (33.4%)		
	OR (95% CI)	Log OR (95% CI)	*P*	OR (95% CI)	Log OR (95% CI)	*P*	OR (95% CI)	Log OR (95% CI)	*P*
**PERCEPTIONS ABOUT HEALTH EFFECTS**									
a. Strongly agree that the personal hair care products I use affect my health.	0.81 (0.37, 1.74)	-0.22 (-0.99, 0.56)	.583	0.89 (0.35, 2.24)	-0.12 (-1.04, 0.81)	.802	0.69 (0.16, 2.98)	-0.37 (-1.84, 1.09)	.619
b. Strongly agree that organic, natural, non-toxic, or eco-friendly personal hair care products have fewer toxic chemicals than regular products.	0.78 (0.50, 1.23)	-0.25 (-0.70, 0.20)	.282	0.76 (0.39, 1.49)	-0.27 (-0.94, 0.40)	.428	0.79 (0.41, 1.53)	-0.24 (-0.90, 0.43)	.483
c. Strongly agree that consumers should be concerned about the health effects of personal hair care products.	1.55 (1.06, 2.27)	0.44 (0.06, 0.82)	.023	2.08 (1.23, 3.52)	0.73 (0.21, 1.26)	.006	1.12 (0.63, 2.02)	0.12 (-0.47, 0.70)	.697
d. Strongly disagree that there is no reason to worry about the health effects from chemicals that might be in personal hair care products.	0.70 (0.47, 1.03)	-0.36 (-0.75, 0.03)	.072	0.50 (0.28, 0.90)	-0.69 (-1.28, -0.11)	.021	1.00 (0.57, 1.74)	0.00 (-0.56, 0.55)	.994
e. Strongly disagree that, overall, the benefits of using personal hair care products outweigh any risks from exposure to toxic chemicals that might be in these products.	1.02 (0.59, 1.77)	0.02 (-0.53, 0.57)	.941	0.72 (0.32, 1.60)	-0.33 (-1.13, 0.47)	.419	1.48 (0.67, 3.26)	0.39 (-0.40, 1.18)	.334
f. Strongly disagree that government agencies in Kenya do a good job of regulating personal care products to ensure they are safe for consumers.	2.06 (1.20, 3.54)	0.72 (0.18, 1.26)	.009	2.10 (0.86, 5.13)	0.74 (-0.15, 1.64)	.104	2.63 (1.25, 5.53)	0.97 (0.23, 1.71)	.011
**PERCEIVED BREAST CANCER RISK**									
a. Strongly agree that I am concerned that the labels of hair care products do NOT list all the ingredients.	1.13 (0.75, 1.71)	0.12 (-0.29, 0.54)	.561	1.34 (0.73, 2.45)	0.29 (-0.32, 0.90)	.347	0.97 (0.53, 1.78)	-0.03 (-0.64, 0.58)	.924
b. Strongly agree that because I am concerned about breast cancer, I plan to go natural (style my hair without chemicals).	0.56 (0.36, 0.86)	-0.59 (-1.02, -0.16)	.008	0.51 (0.29, 0.91)	-0.66 (-1.23, -0.10)	.022	0.64 (0.32, 1.31)	-0.44 (-1.15, 0.27)	.226
c. Strongly agree that because I am concerned about breast cancer, I intend to watch the ingredients of the hair care products I use.	0.92 (0.59, 1.45)	-0.08 (-0.53, 0.37)	.734	1.40 (0.71, 2.74)	0.34 (-0.34, 1.01)	.329	0.64 (0.33, 1.26)	-0.45 (-1.12, 0.23)	.196
d. Strongly agree that all women should worry about the ingredients in hair products.	1.43 (0.92, 2.20)	0.35 (-0.08, 0.79)	.109	1.25 (0.66, 2.38)	0.23 (-0.41, 0.87)	.487	1.65 (0.87, 3.15)	0.50 (-0.14, 1.15)	.128
e. Strongly agree that because I am concerned about breast cancer, I plan to adjust how I use hair care products.	1.67 (1.09, 2.57)	0.51 (0.08, 0.94)	.020	1.40 (0.76, 2.60)	0.34 (-0.28, 0.95)	.280	1.94 (1.03, 3.66)	0.67 (0.03, 1.30)	.040
f. Strongly agree that I want to learn more about the risk hair products can cause to my health.	1.29 (0.84, 1.98)	0.25 (-0.18, 0.68)	.253	1.41 (0.76, 2.63)	0.34 (-0.28, 0.97)	.278	1.06 (0.55, 2.03)	0.06 (-0.59, 0.71)	.864

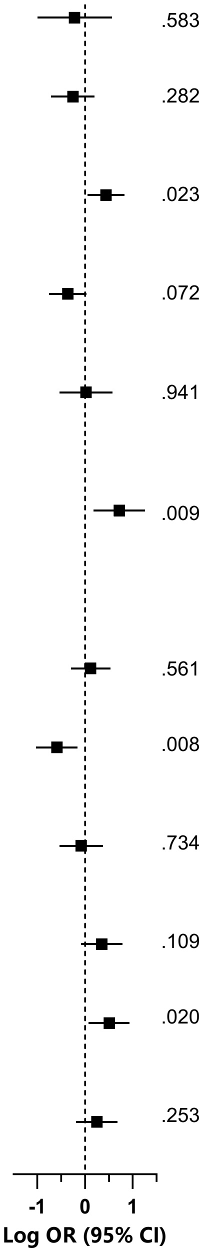

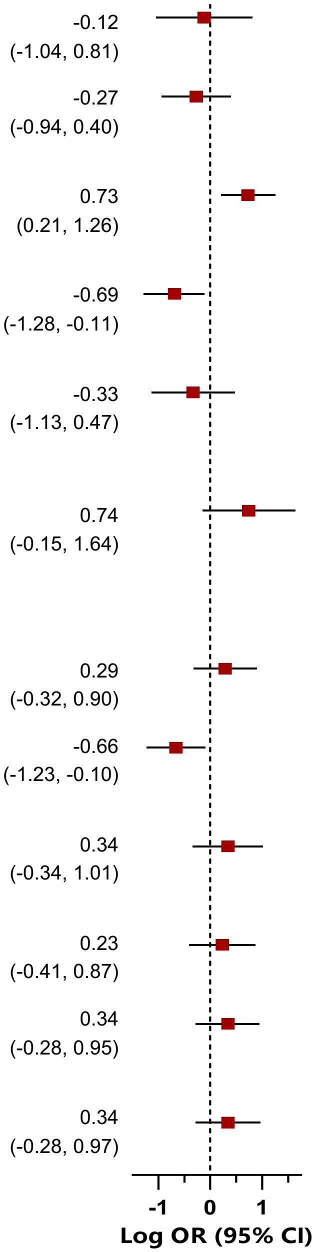

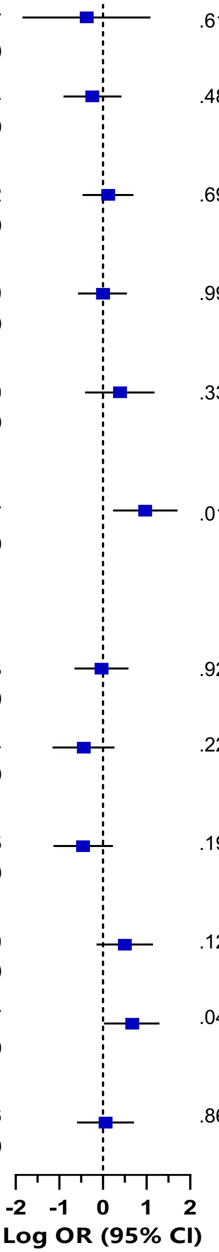

Supplement: Supplementary file 1 [file ijerph-21-00846-s001.zip › ijerph-2997567-supplementary.pdf]
